# Perceived function and physical performance are associated with pain and fatigue in women with fibromyalgia

**DOI:** 10.1186/s13075-016-0954-9

**Published:** 2016-03-16

**Authors:** Dana L. Dailey, Laura A. Frey Law, Carol G. T. Vance, Barbara A. Rakel, Ericka N. Merriwether, Leon Darghosian, Meenakshi Golchha, Katharine M. Geasland, Rebecca Spitz, Leslie J. Crofford, Kathleen A. Sluka

**Affiliations:** Department of Physical Therapy and Rehabilitation Science, Carver College of Medicine, University of Iowa, 1-252 MEB, 500 Newton Road, Iowa City, IA 52242 USA; College of Nursing, University of Iowa, 50 Newton Road, Iowa City, IA 52242 USA; Department of Medicine, Division of Rheumatology & Immunology, Vanderbilt University, 1161 21st Avenue South, Nashville, TN 37232 USA; Department of Physical Therapy and Rehabilitation Science, 1-242 MEB, Carver College of Medicine, University of Iowa, 375 Newton Road, Iowa City, IA 52422 USA

## Abstract

**Background:**

Fibromyalgia is a condition characterized by chronic widespread muscle pain and fatigue and associated with significant impairment in perceived function and reduced physical performance. The purpose of this study was to determine the degree to which pain and fatigue are associated with perceived function and physical performance in women with fibromyalgia.

**Methods:**

Hierarchical linear regression determined the contribution of pain and fatigue (Numeric Rating Scale (NRS) for resting, movement and combined) to perceived function (Fibromyalgia Impact Questionnaire Revised - Function Subscale, FIQR-Function), Multidimensional Assessment of Fatigue - Activities of Daily Living (MAF-ADL) and SF-36 Physical Function Subscale (SF-36-PF) and physical performance (6-Minute Walk Test, 6MWT and Five Time Sit To Stand, 5TSTS) while controlling for age, body mass index, pain catastrophizing, fear of movement, anxiety, and depression in women with fibromyalgia (N = 94).

**Results:**

For perceived function, movement pain and movement fatigue together better predicted FIQR-function (adjusted R^2^ = 0.42, *p* ≤ 0.001); MAF-ADL (adjusted R^2^ = 0.41, *p* ≤ 0.001); and SF-36-PF function (adjusted R^2^ = 0.34, *p* ≤ 0.001). For physical performance measures, movement pain and fatigue together predicted 6MWT distance (adjusted R^2^ = 0.42, *p* ≤ 0.001) and movement fatigue alone predicted performance time on the 5TSTS (adjusted R^2^ = 0.20, *p* ≤ 0.001).

**Conclusions:**

Pain and fatigue are significantly associated with and explain more than one-third of the variance in perceived function and physical performance in women with fibromyalgia.

**Trial registration:**

NIH Clinicaltrials.gov Registration: NCT01888640. Registered 13 June 2013.

## Background

Fibromyalgia is a condition characterized by chronic widespread muscle pain, affecting 4–10 % of the US population, the majority of which are women [1–3]. While pain is the defining characteristic for diagnosing fibromyalgia, fatigue is also a common complaint for individuals with fibromyalgia. For example, Bennett et al. [4] found pain and fatigue emerged as the top two of six distinct clinical features of fibromyalgia in a sample of 788 people with fibromyalgia; 54 % perceived pain as their primary symptom and 28 % perceived fatigue as their main complaint.

That study indicated while pain and fatigue commonly co-occur, they are clearly differentiated in people with fibromyalgia. One of the most challenging aspects of fibromyalgia is the variable nature of pain among individuals with fibromyalgia [5]. Pain for individuals with fibromyalgia may be associated with morning stiffness as well as pain increases throughout the day [6]. Average daily pain ratings may vary in individuals with fibromyalgia with increased pain in the morning and evening [7]. Henriksson found that many people with fibromyalgia describe activities that aggravate the symptoms of fibromyalgia include carrying and holding objects and these activities are perceived as strenuous [8]. Pain in individuals with fibromyalgia has been associated with greater disease severity, reduced function and symptoms of fibromyalgia [9, 10]. Thus, pain is a significant symptom that may contribute to participation in physical function.

The perception of fatigue is a significant symptom that, in addition to pain, may contribute to reduced participation in daily physical activities. Although fatigue is not part of the American College of Rheumatology (ACR) clinical definition published in 1990, it is taken into consideration as part of the newer criteria published in 2010 [11, 12]. Fatigue is a major symptom that impacts function and is reported in nearly 100 % of the population [11, 13] People with fibromyalgia typically describe their physical fatigue as an overall feeling of tiredness or exhaustion [2], as a failure to initiate or sustain physical activities [14], or having to take things slower in order to accomplish tasks [15]. Individuals with fibromyalgia frequently report difficulty with daily functional tasks (e.g., pouring a cup of coffee, folding laundry, drying hair or getting dressed), [2, 15] strenuous physical activities, and cognitive tasks such as paying bills [15].

Function is significantly impaired in people with fibromyalgia with poor perceived function observed on several self-report measures [4, 16–21]. Physical performance on clinical tests, such as the 6-Minute Walk Test (6MWT) or Five Time Sit To Stand (5TSTS) test, is also significantly reduced in this population when compared to healthy controls [22–24] Perceived function does not directly assess physical performance, and self-report and physical performance are not strongly correlated [25, 26]. Clinically, people with fibromyalgia report that both pain and fatigue are major barriers to their ability to participate in regular activities [2, 15]. Pain and fatigue during functional activities, particularly movement pain and movement fatigue, may be greater barriers to participation in these activities and are significantly increased in people with fibromyalgia during fatiguing exercise tasks [25, 27, 28]. However, the degree to which pain and fatigue diminish perceived function and physical performance has not been well investigated.

Accordingly, the primary purpose of this study was to determine the degree to which self-reported pain and fatigue intensities are associated with perceived function and physical performance in women with fibromyalgia. We hypothesized that higher levels of pain and fatigue would be associated with reductions in perceived function and physical performance, with pain and fatigue intensity each contributing unique information to the model. A secondary purpose was to investigate whether pain and fatigue assessed during rest, movement or the change between rest and movement would best predict perceived function and physical performance.

## Methods

This study is a supplementary analysis using baseline data from a clinical trial, Fibromyalgia Activity Study with TENS (FAST) [29] before randomization to a Transcutaneous Electrical Nerve Stimulation (TENS) treatment. Following completion of written consent, demographic information was gathered for the participants with respect to age, sex, ethnicity, marital status, education, income, body mass index (BMI) and length of diagnosis of fibromyalgia (Table 1). Internal consistency was calculated for measures of pain, fatigue, physical performance, perceived function and control variables (Tables 1 and 2). Additional details related to the parent study, including subject inclusion and exclusion criteria, measures used in this analysis, and their justification for use in the study have been reported previously [29].

**Table 1:** Subject characteristics at baseline visit (N = 94 women)

Variable	Mean ± SD or N (%)	Range	Cronbach's α
Age (years)	49.2 ± 11.0	20 to 67	
BMI	33.8 ± 7.3	19.1 to 70.2	
Caucasian	88 (93.6 %)	-	
Marital status:			
Married/cohabitating	46 (48.9 %)	-	
Single/widowed/divorced	46 (48.9 %)	-	
No answer	2 (2.1 %)	-	
Education:			
High school or less	24 (25.5 %)	-	
Some college or more	66 (70.2 %)	-	
No answer	4 (4.3 %)	-	
Annual income:			
< $60,000	60 (63.8 %)	-	
≥ $60,000	29 (30.9 %)	-	
No answer	5 (5.3 %)	-	
Years since diagnosis:			
< 10 years	53 (56.4 %)	-	
10–19 years	31 (33 %)	-	
≥ 20 years	10 (10.6 %)	-	
Comorbidities (0–15)	1.78 ± 0.15	0 to 7	
Cancer	54 (57.44 %)	-	
Heart disease	28 (29.7 %)	-	
OA	25 (26.5 %)	-	
PCS (0-52)	20.1 ± 13.2	0 to 51	0.95
PROMIS Anxiety (T-score)	57.9 ± 7.9	37.1 to 83.1	0.93
PROMIS Depression (T-score)	57.5 ± 8.4	37.1 to 81.1	0.93
TSK (0-68)	35.9 ± 7.9	18 to 58	0.84

*Abbreviations*: *SD* standard deviation, *BMI* body mass index, *OA* osteoarthritis, *PCS* Pain Catastrophizing Scale, *PROMIS* National Institutes of Health Patient Reported Outcome Measurement Information System, *TSK* Tampa Scale of Kinesiophobia, *T-score* (mean of 50 with SD of 10)

**Table 2:** Summary of pain, fatigue, and function scores (N = 94)

Variable	Mean ± SD	Range	Cronbach's α
Pain rest (0–10)	6.0 ± 1.6	2 to 10	0.53
Pain movement (0–10)	6.4 ± 1.9	2 to 10	
Pain change score	0.3 ± 1.7	(-5.5) to 6	
Fatigue rest (0–10)	6.5 ± 2.0	2 to 10	0.54
Fatigue movement (0–10)	5.6 ± 2.5	0 to 10	
Fatigue change score	(-0.9) ± 2.4	(-8) to 5	
FIQR-Function (0–30)	15.1 ± 5.8	1.3 to 28.3	0.89
MAF-ADL (0–10)	5.8 ± 1.8	2.1 to 10	0.86
SF-36-PF (T-score)	33.4 ± 7.5	19.3 to 49.9	0.84
6MWT (feet)	1313.6 ± 323.5	100 to 2000	-0.042
5TSTS (seconds)	13.4 ± 6.6	6 to 51.5	

*Abbreviations*: *SD* standard deviation, *FIQR-Function* Fibromyalgia Impact Questionnaire Revised - Function Subscale, *MAF-ADL* Multidimensional Assessment of Fatigue - Activities of Daily Living, *SF-36-PF* Short-Form Health Survey Physical Function Subscale, *6MWT* 6-Minute Walk Test, *5TSTS* Five Time Sit to Stand, *T-score* (mean of 50 with SD of 10), and *change scores*: movement – resting scores

### Subjects

Following approval by the Institutional Review Boards at both study sites, 94 women with fibromyalgia were recruited from the regions surrounding the University of Iowa Hospitals and Clinics and Vanderbilt University. Inclusion criteria for the individuals with fibromyalgia included: (1) females ages 18–70 years, (2) diagnosis of fibromyalgia confirmed with ACR 1990 criteria with 11 of 18 tender points (TeP) with TeP examination, (3) pain greater than or equal to 4/10 at time of telephone screen, (4) history of cervical or lumbar pain, (5) current stable treatment regimen for the last 4 weeks and projected stable treatment regimen for the next 2 months, and (6) ability to speak English. Subjects were excluded if they had: (1) current or history of cardiovascular, pulmonary, neurological, endocrine, or renal disease that would preclude involvement in the study, (2) TENS use in the last 5 years, (3) pacemaker, (4) uncontrolled blood pressure or diabetes, (5) neuropathic pain condition, (6) systemic autoimmune disorder, (7) cervical or lumbar fusion or metal implants, (8) severe skin allergy to adhesive, (9) allergy to nickel, (10) pain less than 4, (11) pregnancy, (12) epilepsy, (13) change in treatment program (pharmacological or nonpharmacological) within the last month or planned for the next 2 months, (14) unstable medical or psychiatric condition which in the opinion of the investigator could compromise the subject’s welfare or confound the study results, (15) chest pain with activity such as walking or climbing stairs, and (16) ambulation with assistive device, e.g., cane or walker.

### Pain intensity measure

An 11-point Numeric Rating Scale (NRS) was used for measurement of pain intensity at rest and with movement (during 6MWT). The pain NRS scale was vertical with anchors at the bottom, “no pain,” and top, “worst pain imaginable”. Pain NRS has adequate test-retest reliability (r = 0.63) [30].To examine the impact of movement on pain we calculated each individual’s ‘pain change’ score as movement minus resting pain.

### Fatigue intensity measure

An 11-point (0–to 10) NRS was used to measure perceived fatigue intensity at rest and with movement (during a 6MWT, see below). The fatigue NRS scale was vertical with anchors at the bottom, “no fatigue,” and top, “worst fatigue imaginable”. The psychometric properties of this fatigue NRS have not been determined. However, similar NRS scales for pain have established reliability and validity as discussed above [31]. To examine the impact of movement on fatigue intensity we calculated each individual’s ‘fatigue change’ score as movement fatigue intensity minus resting fatigue intensity.

### Self-report function measures

#### Fibromyalgia Impact Questionnaire Revised (FIQR)

The FIQR is a 21-item standard measure used as an index of fibromyalgia disease activity and impact over the past 7 days [18]. Subjects are asked to indicate the degree to which fibromyalgia has impacted multiple domains. The three domains of the FIQR include function (nine items), symptoms (ten items) and overall impact (two items). For this study, only the function domain was used as a measure of disease-specific perceived function. The nine-function questions ask the subject to score the level of difficulty (0–10 scale from “no difficulty” to “very difficult”) with the following tasks: brush or comb your hair; walk continuously for 20 minutes; prepare a homemade meal; vacuum, scrub or sweep floors; lift and carry a bag full of groceries; climb one flight of stairs; change bed sheets; sit in a chair for 45 minutes; and go shopping for groceries. Each of the nine items is scored 0–10, summed, and then divided by three for a possible score of 0–30, where a higher score indicates greater perceived functional difficulty. The FIQR-Function domain demonstrates convergent validity when compared to SF-36 physical function (SF-36-PF) subscale (r = 0.80, *p* < 0.001) [18].

#### Multidimensional Assessment of Fatigue (MAF)

The MAF is a 16-item self-report measure of fatigue used in chronic illness that can be subdivided into four dimensions: severity, distress, timing (over the past week, when it occurred and any changes), and impact on various activities of daily living (ADL) (household chores, cooking, bathing, dressing, working, socializing, sexual activity, leisure and recreation, shopping, walking, and exercising). We used the ADL subscale of the MAF as a fatigue-specific measure of self-reported function, where a higher score indicates greater perceived functional difficulty with ADLs. Psychometric properties for the ADL subscale have not been established. The MAF has been shown to have internal consistency r = 0.93; and convergent validity with a fatigue Visual Analogue Scale (VAS) r = 0.80, *p* < 0.05 [32].

#### SF-36

The SF-36 is a commonly used survey of self-reported patient health. It has 36 questions with eight sections: vitality, physical functioning, bodily pain, general health perceptions, physical role functioning, emotional role functioning, social role functioning and mental health [33]. We used the physical functioning subscale as a measure of global self-reported function (not disease- or symptom-specific). It consists of ten items, inquiring if health limits physical activity, basic mobility and basic activities of daily living on a 4-point scale: yes, limited a lot; yes, limited a little; and no, not limited at all. The SF-36 raw data is rescaled to a T-score with a mean of 50 and a standard deviation (SD) of 10 with higher scores demonstrating better health. The physical function subscale raw score is also converted to a T-score with a mean of 50 and a SD of 10 (lower scores indicate worse function). The SF-36-PF Subscale has internal consistency of 0.62 to 0.96 and test-retest reliability of 0.42 to 0.90 [33].

### Physical performance measures

#### Six-Minute Walk Test (6MWT)

Subjects completed the 6MWT using a 100-foot lap with a turn at the 50-foot mark. Pain and fatigue ratings (NRS) were completed at the 5-minute mark The 6MWT is a standard function test that measures the maximum distance (feet) a person can walk as fast as is comfortable in 6 minutes. NRS pain and fatigue intensities were rated near the end of the task for ratings of movement pain and movement fatigue, respectively. The 6MWT is a submaximal test of aerobic capacity with indications for endurance [34]. The 6MWT has excellent test-retest reliability (intraclass correlation coefficient (ICC) = 0.95–0.97) and construct validity (r = 0.63–0.79) [34].

#### Five Time Sit to Stand Test (5TSTS)

The 5TSTS is a test of lower body strength. Subjects completed the test with five repetitions of sit to stand transitions, completed as quickly as possible. The time to complete five repetitions was recorded. Pain and fatigue ratings (NRS) were completed at the end of the test. The time it takes to complete five repetitions of sit to stand, completed as quickly as possible, is recorded. The 5TSTS has adequate test-retest reliability (ICC > 0.83) [35, 36].

#### Psychological measures

Pain catastrophizing, fear of movement, anxiety and depression are psychological factors that can influence pain, fatigue, and function [37]. Thus, we assessed and adjusted our analyses for the following psychological variables:

#### Pain Catastrophizing Scale (PCS)

The PCS is a 13-item questionnaire regarding feelings and thoughts related to the experience of pain. The PCS has demonstrated internal consistency with Cronbach's alpha = 0.95 and criterion related validity with Cronbach's alpha = 0.42 [38].

#### Fear of movement (Tampa Scale of Kinesiophobia, TSK)

The TSK is a 17-item measure of fear of movement or re-injury that is used in patients with chronic pain considering multiple scenarios (e.g., physical and work activity). Test-retest reliability is good with r = 0.78 and good internal consistency with Cronbach's alpha equal to 0.76 [39, 40].

#### PROMIS Short Form 8a Anxiety and 8b Depression

The NIH Patient Reported Outcome Measurement Information System (PROMIS) Short Forms assess universal rather than disease-specific questions regarding anxiety (eight items) and depression (eight items) in the last 7 days. Each scale consists of eight questions scored from 1 to 5 for a total raw score ranging from 8 to 40. The raw scores are converted to separate T-scores for comparison of the anxiety and depression scales. The T-score rescales each raw score into a standardized score based on a standard population with a mean of 50 and a standard deviation (SD) of 10 [41]. A score higher than 50 represents greater anxiety or depression and a score lower than 50 represent less anxiety or depression. The Anxiety scale measures self-reported fear, anxious misery, hyperarousal and somatic symptoms related to arousal [41]. The Depression scale measures negative mood, views of self, social cognition and decreased positive affect and engagement [41]. Psychometric properties for the eight- item Short Form have not been established. However the four-item Short Forms for anxiety and depression demonstrate good internal consistency with Cronbach's alpha of 0.89 ± 1.24 standard error of measure (SEM) and 0.93 ± 1.08 (SEM), respectively [42].

### Data analysis

Normality was assessed utilizing Kolmogorov-Smirnov test. Descriptive statistics (mean, standard deviation) were calculated for clinical characteristics and outcome variables. The relationships between individual variables were assessed using Pearson’s correlation coefficients and correlations r < 0.70 was utilized for inclusion in the analysis. In particular, the associations between the control variables, the predictor variables and the outcome variables were assessed separately. Internal consistency of variables were calculated and represented with Cronbach’s alpha in Tables 1 and 2.

The relationships between pain and fatigue with our primary outcome variables of perceived function (FIQR-Function, MAF-ADL and SF-36-PF) and secondary outcome variables of physical performance (6MWT, 5TSTS) were explored using hierarchical linear regression analyses (30 calculations were completed). We first included demographic (age and BMI) and psychological variables (pain catastrophizing, fear of movement, anxiety, depression) in our base regression models to understand the contributions of these control variables in predicting our outcome variables (step 1). Then separately, either pain (resting pain, movement pain, or pain change, step 2A) or fatigue (resting fatigue, movement fatigue, or fatigue change, step 2B) variables were added to the base models to examine how either pain or fatigue alone would improve the fit of the regression models in predicting perceived function or physical performance over the control variables. These are referred to as the single-predictor models, serving as intermediate steps in our hierarchical regression. Lastly, both pain and fatigue (resting, movement, or change) variables were included in the final step (step 3) for each of the five outcome variables. This allowed us to determine the degree to which accounting for both pain and fatigue improved the regression model fits beyond the single-predictor models which only included either pain or fatigue in isolation. For each step, adjusted R^2^ values were calculated, reflecting the proportion of variance in the outcome variable explained by the model, adjusted for the number of predictors included in the model. The significance of the change in R^2^ (ΔR^2^) between successive steps was assessed to determine whether the addition of pain or fatigue variables resulted in a significant improvement in predicting each outcome variable. To determine if the combination of pain and fatigue proved to be significantly better at predicting the outcomes than either pain or fatigue alone, the step 3 models were compared to the better of the two single-predictor (step 2A or 2B) models. To address our secondary aim, comparing resting, movement, and change scores for pain and fatigue, the models’ R^2^ values using each form of assessment at each step in the hierarchical regression were compared. Because of the potential outlier BMIs in the study we repeated the analysis with the removal of the single highest BMI (70.19), and removal of the highest three BMI values (52.61, 70.19, 54.24). The regression models with and without these potential outliers did not significantly change. Thus, we report the data for the complete set only for brevity. The Statistical Package for the Social Sciences (IBM SPSS v.21, IBM Corp., Armonk, NY, USA) was used for all statistical analyses; significance was set at *p* ≤ 0.05 and *p* values are provided for each test.

## Results

### Clinical characteristics

Ninety-four women with fibromyalgia with an average age of 49.2 years (range 20–67) and BMI of 33.8 kg/m^2^ (range 19.1–70.2) participated in the study (Table 1). The majority of the subject sample was Caucasian (93.6 %), had an income less than $60,000 per year (63.8 %) and attained an education level of some college or more (70.2 %). Approximately half were married or cohabitating (48.9 %) and half were single, widowed, or divorced (48.9 %). There was a wide range of scores on the psychological factors from within normal range to the extreme range for pain catastrophizing, fear of movement, anxiety and depression. Comorbidities were collected and the three comorbidities that occurred in greater than 25 % of the sample were included in Table 1.

Resting pain intensity in this sample averaged 6.0 ± 1.6 (mean ± SD), while movement pain intensity averaged 6.4 ± 1.9 (mean ± SD) (Table 2). The change in pain intensity from resting to during movement averaged 0.3 ± 1.7, with a wide range of responses from a decrease in intensity of up to 5.5/10 to an increase of up to 6/10. Similarly, resting fatigue intensity averaged 6.5 ± 2.0 and movement fatigue intensity averaged 5.6 ± 2.51. The change in fatigue intensity from resting to during movement ranged from a decrease of 8 to an increase of 5, with an average decrease in fatigue intensity rating after performing the 6MWT (-0.9 ± 2.4). Thus, some people showed a reduction in pain and fatigue intensity with movement while others showed increases.

Fibromyalgia subjects showed significant impairments in perceived function and physical performance (Table 2). The FIQR-Function scale averaged 15.1 ± 5.8 (on a 0 to 30 scale), the MAF-ADL subscale averaged 5.8 ± 1.8 (0–10), and the SF-36-PF averaged 33.4 ± 7.5 (T-score with 50 average). For physical performance tasks, subjects walked 1313.6 ± 323.5 feet on the 6MWT (1876 feet is normal in 60- to 69-year-old females [34]) and took 13.4 ± 6.6 seconds to complete the 5TSTS (11.4 seconds normal for age 60–69 years [34]).

### Correlation analysis

The psychological traits were significantly correlated with the control variables (Table 3). Age and BMI were not correlated with any of the other control variables. Anxiety, depression, and pain catastrophizing were strongly correlated with each other (r = 0.60 – 0.66). Fear of movement moderately correlated with pain catastrophizing (r = 0.47) and anxiety (r = 0.39) and weakly correlated with depression (r = 0.28).

**Table 3:** Pearson correlation coefficients between control variables

	Age	TSK	PCS	Anxiety	Depression
BMI (kg/m^2^)	0.157	-0.067	-0.16	-0.13	-0.085
Age (years)		-0.124	-0.088	-0.08	0.003
Fear of movement (TSK)			**0.473** **	**0.385** **	**0.282** **
Pain catastrophizing (PCS)				**0.605** **	**0.598** **
Anxiety					**0.663** **

*Abbreviations*: *BMI* body mass index, *TSK* Tampa Scale of Kinesiophobia, *PCS* Pain Catastrophizing Scale

**p ≤ 0.01 level, *p ≤ 0.05 level

Resting and movement pain and fatigue were moderately correlated with each other (Table 4). The change scores were consistently positively correlated with movement assessments, and negatively correlated with resting assessments (the higher the resting score, the lower the change with movement). Movement pain and fatigue from the 6MWT was moderately correlated with the movement pain and fatigue from the 5TSTS. Since 6MWT was performed first and is a common test in a variety of populations, movement pain and fatigue from the 6MWT was used to represent movement pain and fatigue in the analysis.

**Table 4:** Pearson correlation coefficients between pain and fatigue predictor variables

	Pain movement	Pain change	Fatigue rest	Fatigue movement	Fatigue change
Pain rest	**0.505** **	**-0.363** **	**0.589** **	0.186	**-0.312** **
Pain movement		**0.621** **	**0.512** **	**0.450** **	-0.002
Pain change			0.019	**0.313** **	**0.281** **
Fatigue rest				**0.434** **	**-0.367** **
Fatigue movement					**0.679** **

**p ≤ 0.01 level, *p ≤ 0.05 level

Perceived function measures (FIQR-Function, MAF-ADLs and SF-36-PF) and physical performance measures (6MWT, 5TSTS) were all weakly (r = 0.30) to moderately (r = 0.66) correlated (Table 5). The highest correlations were between the three perceived function measures, FIQR-Function, MAF-ADL, and SF-36-PF, with SF-36-PF score inversely related to the FIQR-Function and MAF-ADL scores due to the reverse scaling of that instrument. Similarly, the physical performance assessments (6MWT and 5TSTS) were weakly to moderately correlated with the three perceived function assessments (Table 5).

**Table 5:** Pearson correlation coefficients between function outcome variables

	MAF-ADL	SF-36-PF	6MWT	5TSTS
FIQR-Function	**0.617** **	**-0.655** **	**-0.405** **	**0.426** **
MAF-ADL		**-0.533** **	**-0.313** **	**0.301** **
SF-36-PF			**0.457** **	**-0.314** **
6MWT				**-0.534** **

*Abbreviations*: *MAF-ADL* Multidimensional Assessment of Fatigue - Activities of Daily Living, *SF-36-PF* Short-Form Health Survey Physical Function Subscale, *6MWT* 6-Minute Walk Test, *5TSTS* Five Time Sit to Stand, *FIQR-Function* Fibromyalgia Impact Questionnaire Revised - Function Subscale

**p ≤ 0.01 level, *p ≤ 0.05 level

### Perceived function

The results of the hierarchical linear regression models for the three perceived function measures are provided in Table 6. The control variables in step 1, i.e., age, BMI, and psychological factors, significantly predicted from 11 % to 22 % of the variance in perceived function (Table 6). The addition of either pain or fatigue (steps 2A or 2B) provided a significant improvement over the base model for the majority of the three perceived function outcomes. Change in pain (step 2A) was not better than the control variables alone for FIQR-Function (*p* = 0.12). The addition of resting pain to the control variables did not help explain the MAF-ADL (*p* = 0.11). For the models including a fatigue predictor (step 2B), the change score never improved the model predictions of perceived function over the control variables (*p* = 0.23 – 0.33).

**Table 6:** Hierarchical linear regression models for pain, fatigue, and pain and fatigue predicting the perceived function outcome variables, FIQR-Function, MAF-ADL, and SF-36-PF, controlling for age, BMI, pain catastrophizing, fear of movement, anxiety, and depression

		FIQR-Function			MAF-ADL			SF36-PF		
		Adj. R^2^	Change (Δ) in R^2^	*p* value R^2^ change	Adj. R^2^	Change (Δ) in R^2^	*p* value R^2^ change	Adj. R^2^	Change (Δ) in R^2^	*p* value R^2^ change
Step 1	Control variables	**0.13** **			**0.22** **			**0.11** ^*^		
Step 2A: pain	Resting	**0.29** **	0.16	**<0.001**	0.23**	0.01	0.11	**0.17** **	0.06	**0.01**
	Movement	**0.37** **	0.26	**<0.001**	**0.32** **	0.1	**<0.001**	**0.33** **	0.22	**<0.001**
	Change	0.15**	0.02	0.12	**0.26** **	0.04	**0.02**	**0.18** **	0.07	**0.007**
Step 2B: fatigue	Resting	**0.29** **	0.16	**<0.001**	**0.30** **	0.08	**0.002**	**0.19** **	0.08	**0.004**
	Movement	**0.31** **	0.18	**<0.001**	**0.39** **	0.17	**<0.001**	**0.22** **	0.03	**0.001**
	Change	0.13**	0	0.23	0.25**	0.03	0.33	0.12*	0.01	0.27
Step 3: pain and fatigue	Resting	**0.32** **	0.11	**0.03**	0.29**	0.03	0.76	0.19**	0.08	0.28
	Movement	**0.42** **	0.29	**0.006**	**0.41** **	0.19	**≤0.001**	0.34**	0.23	0.1
	Change	0.14**	0.01	0.49	0.27**	0.05	0.15	0.17**	0.06	0.83

Control variables: age, body mass index (BMI), pain catastrophizing (PCS), fear of movement (TSK), anxiety (PROMIS Anxiety), and depression (PROMIS Depression). Resting, movement, and change scores entered into separate regression models for pain (step 2A), fatigue (step 2B), and pain and fatigue together (step 3).

*Abbreviations*: *FIQR-Function* Fibromyalgia Impact Questionnaire Revised - Function Subscale, *MAF-ADL* Multidimensional - Assessment of Fatigue Activities of Daily Living, *SF-36-PF* Short-Form Health Survey Physical Function

R^2^ significance level: **p ≤ 0.01 level; *p ≤ 0.05 level

Supporting our initial hypothesis, including both movement pain and fatigue variables in the regression models (step 3) consistently resulted in significantly better predictions of perceived function based on the FIQR and MAF-ADL, indicating both pain and fatigue uniquely contribute to these outcomes than either one alone. For the MAF-ADL, the addition of resting pain (step 3) was not better than the model relying on resting fatigue alone (step 2B). The perceived function assessed by the SF-36 was approximately equally well predicted by either pain or fatigue alone, but no significant improvement was seen by including both pain and fatigue in the model.

Overall, self-reported movement pain and fatigue intensities explained a higher proportion of the variance observed in all three perceived function assessments (FIQR-Function, MAF-ADL, and SF-36-PF) than self-reported resting pain and fatigue intensities (Table 6). This was most notable for the resting pain scores as resting fatigue typically explained a greater proportion of perceived function than resting pain. However, movement pain intensity was often superior to movement fatigue intensity when they were considered in isolation (see Table 6). The change scores for pain or fatigue intensities were not strongly associated with perceived function. Only two of the nine possible models including a change score provided significantly more predictive value than the lower level corresponding model: change in pain provided significantly more information than the control variables alone for MAF-ADL and SF-36-PF. Overall, movement pain and movement fatigue intensities together explained from 34 % to 42 % of the variance in perceived function, after controlling for age, BMI, pain catastrophizing, fear of movement, anxiety and depression, an increase of 19 to 29 % of the variance explained compared to the control variables alone.

### Physical performance

The results of the hierarchical linear regression models for the two physical performance measures are provided in Table 7. The control variables in step 1, i.e., age, BMI, and psychological factors, predicted 31 % of the variance in the 6MWT, but only 5 % of the 5TSTS. The addition of movement pain (step 2A) provided a significant improvement over the base model for either 6MWT or 5TSTS. Resting pain and pain change did not significantly account for variance in 6MWT or 5TSTS. Both resting and movement fatigue (step 2B) also provided a significant improvement in predicting physical performance over the control variables alone (step 1), but movement fatigue produced a larger improvement than resting fatigue. Change in pain or fatigue scores did not significantly improve the models, except for predicting the 5TSTS, which was similar to the resting fatigue model. Including both pain and fatigue in the regression models (step 3) did not result in better predictions of physical performance compared to either fatigue or pain alone (steps 2A or 2B), contrary to our initial hypothesis.

**Table 7:** Hierarchical linear regression predicting the function outcome variables, 6-Minute Walk Test (6MWT) and Five Time Sit To Stand (5TSTS), controlling for age, BMI, pain catastrophizing, fear of movement, anxiety, and depression

		6MWT			5TSTS		
		Adj. R^2^	Change (Δ) in R^2^	*p* value R^2^ change	Adj. R^2^	Change (Δ) in R^2^	*p* value R^2^ change
Step 1	Control variables	**0.31** **			0.05		
Step 2A: pain only	Resting	**0.35** **	0.04	0.26	0.05	0	0.34
	Movement	**0.40** **	0.09	**0.001**	**0.08** *	0.03	**0.05**
	Change	**0.33** **	0.02	0.1	0.06	0.06	0.25
Step 2B: fatigue	Resting	**0.35** **	0.04	**0.01**	**0.10** *	0.05	**0.03**
	Movement	**0.38** **	0.07	**0.001**	**0.20** **	0.15	**<0.001**
	Change	**0.32** **	0.01	0.17	**0.10** *	0.05	**0.02**
Step 3: pain and fatigue	Resting	**0.35** **	0.02	0.35	**0.09** *	0.04	0.62
	Movement	**0.42** **	0.11	0.17	**0.20** **	0.15	0.71
	Change	**0.33** **	0.02	0.39	0.10*	0.05	0.71

Control variables: age, body mass index (BMI), pain catastrophizing (PCS), fear of movement (TSK), anxiety (PROMIS Anxiety), and depression (PROMIS Depression)

R^2^ significance level: **p ≤ 0.01 level; *p ≤ 0.05 level

Consistent with perceived function models, self-reported movement pain or fatigue intensities were consistently better than resting pain or fatigue intensities. Movement pain and movement fatigue intensities explained 40 % and 38 % of the variance in the 6MWT, respectively. Thus, the 42 % of the variance explained by the model including both movement pain and fatigue intensities was not significantly better than the individual scores. For the 5TSTS, movement fatigue intensity was a better predictor of performance than movement pain intensity (20 % vs 8 % variance explained by each respective model), and the inclusion of both did not significantly improve the variance explained (20 %).

Overall, movement pain and movement fatigue intensities together or in isolation were able to explain from 20 % to 42 % of the variance in physical performance, after controlling for age, BMI, pain catastrophizing, fear of movement, anxiety and depression. This represents an increase of 11 to 15 % of the variance explained compared to the control variables alone.

## Discussion

The current study shows that both pain and fatigue intensities are significant contributors to perceived function and physical performance. Using different perceived function measures and physical performance measures we show different contributions of pain and fatigue supporting that they are unique constructs affecting perceived function and physical performance Interestingly, in the current study there was a wide variation in the pain and fatigue response to movement with some showing increases and others no change or a decrease, suggesting a heterogeneous response to movement. In our models, pain and fatigue, along with demographic and psychological variables, explain up to 43 % of the variance observed in perceived function and up to 41 % of the variance observed in physical performance.

### Perceived function

Perceived function has previously been measured in women with fibromyalgia with a variety of self-report measures [43, 44]. In agreement with the current study, Wolfe et al. found that the primary determinants of global severity and disease impact were pain, fatigue and function in individuals with fibromyalgia [13] In addition to the FIQR and SF-36, we also examined the MAF-ADL as an additional measure of perceived function. Each tool provides a slight variation on perceived fatigue. The FIQR is a disease-specific measure of perceived function. The SF-36 is used across a wide range of patient populations as well as healthy control populations [44] making it a general measure of perceived function. The MAF-ADL provides an alternate assessment of perceived function with a greater emphasis on the impact of fatigue. Our findings extend the results from prior studies by examining the unique and distinct influences of pain and fatigue on evaluating perceived function using multiple related, yet unique, measures.

Both the FIQR-Function and MAF-ADL were better represented by the inclusion of both pain and fatigue intensities in the model. The inclusion of pain and fatigue did not better explain the SF-36-PF. However, when pain or fatigue were considered in isolation, the FIQR-Function and SF-36-PF were equally well represented by either pain or fatigue, suggesting similar degrees of influence on these measures of perceived function. Conversely, for the MAF-ADL, fatigue was a better predictor than pain alone, consistent with its development as a tool to assess the impact of fatigue. Despite this, pain provided additional information in predicting the MAF-ADL, beyond fatigue alone. Thus, pain and fatigue are related to all three assessments of perceived function, particularly movement pain and movement fatigue.

Psychological and demographic variables also contributed to perceived function. We found 11–22 % of the variance in perceived function was explained by a combination of age, BMI, and psychological traits. Pain catastrophizing was associated with duration of fibromyalgia diagnosis (r = 0.27–0.73, *p* < 0.05) [45], and predicted perception of pain better than age or education [46]. Prior studies also show that anxiety and depression contribute to decreased perceived function and increased disease impact [47, 48]. The relationship between fear of movement and perceived function in individuals with fibromyalgia had not been previously studied. The associations observed between these variables in our cohort support that anxiety, depression, catastrophizing and fear are significantly related, and collectively influence perceived function in women with fibromyalgia.

### Physical performance

Few studies have examined physical performance in individuals with fibromyalgia, and fewer still have examined factors associated with physical performance. We examined, for the first time, the association of pain and fatigue with two performance measures, the 6MWT and 5TSTS and show movement pain is associated with performance on 6MWT while resting and movement fatigue is associated with performance on the 5TSTS. In agreement with our study, a small study (n = 18) showed higher self-reported pain on the FIQ correlated with reduced walking distance in the 6MWT [49]. Previous studies suggest that the 6MWT is a measure of endurance and the 5TSTS is a measure of strength [34, 50, 51] and are therefore thought to be different measures of performance. Our data confirm these measures are distinct and suggest pain has more influence on endurance whereas fatigue is more closely associated with strength. However, fatigue and endurance are inherently related constructs [13], thus this finding is somewhat unexpected. We cannot rule out that this difference may be a result of the relatively small variance explained in the 5TSTS overall (20 %) relative to the 6MWT (42 %). The control variables, including age, BMI, and psychological traits, explained only 5 % of the variance in the 5TSTS, whereas 31 % of the variance in the 6MWT was explained by the control variables. These data support our proposal that physical performance measures are uniquely different from perceived function and thus provide a useful additional outcome measure to examine function in people with fibromyalgia.

### Fibromyalgia and fatigue

Few studies have focused on fatigue in people with fibromyalgia despite the fact that the majority of people with fibromyalgia report fatigue in a similar frequency to pain [52, 53]. Indeed we show that fatigue is a common problem in this sample, averaging 6.5 on a 10-point scale. Further fatigue played a significant role in perceived function and physical performance. Our results support the inclusion of movement fatigue measures, in addition to movement pain measures, in future studies involving individuals with fibromyalgia since fatigue also influences physical performance and perceived function.

### Fibromyalgia and pain

A number of studies have examined predictors of pain in individuals with fibromyalgia. These studies in general show that psychological factors, quantitative sensory tests, and clinical characteristics (age, BMI) contribute to the severity of pain [1, 54, 55]. Specifically, in people with fibromyalgia (n = 74), anxiety was most closely associated with the sensory dimension of pain, helplessness was most closely associated with the affective dimension of pain, and fear of pain was closely associated with pain intensity [47]. Further, chronic widespread pain was predicted by the number of body pain areas, negative affect, heat, temporal summation of pain and ratings of “after sensations” [1, 54]. Greater physical pain was reported by women with fibromyalgia who were older, less educated, more depressed, and had higher BMI and those with osteoarthritis (n = 238) [55]. Interestingly, a combination of tender point counts, negative affect and wind-up accounted for pain after sensations contributing up to 27 % [1, 54]. Thus, multiple factors impact pain intensity experienced with fibromyalgia including the number of body pain areas, psychological constructs, and response to peripherally applied stimuli. The current study, on the other hand, showed pain severity contributes to perceived function and physical performance.

### Limitations

While our intent was to assess perceived function and physical performance, we recognize the physical performance measures assessed (6MWT, 5TSTS) may not fully represent an individual’s function at home, work or in social situations. These assessments are commonly used to identify function in research and clinical practice settings using a standardized protocol, but may differ from an individual’s daily function. Another limitation in our study is the association between pain and fatigue that may limit the ability of regression models to fully assess their relative importance in predicting function. However, the use of hierarchical models allowed us to examine the contribution of each, limiting the influence of colinearity on our conclusions. Another limitation is the fact that we had a number of exclusion criteria, including male sex, which may limit some generalizability. In our sample, subjects had an average of 1.78 of 15 comorbidities with a range of 0 to 7. Finally, our analysis did not include all possible relevant predictors to perceived function and physical performance; the amount of variance explained suggests other factors also contribute to these outcomes. Future studies would benefit from the inclusion of sleep, physical activity levels and self-efficacy as these factors have been shown to contribute to fatigue and pain in fibromyalgia [56, 57], and thus could contribute to physical performance and perceived function.

## Conclusions

The impact of pain and fatigue intensities is an important consideration in the assessment and treatment of individuals with fibromyalgia in a clinical setting. While these two constructs are clearly related, our findings indicate that pain and fatigue each have independent contributions to perceived function and physical performance. Further, assessment of pain and fatigue with movement, when compared to rest, shows greater associations with function. Thus, these findings suggest it would be important to assess both pain and fatigue intensities during clinical and functional activities and the timing of these assessments (before and during activities) in the clinic and at home may be important. Clinically, pain and fatigue may adversely impact patient performance, participation and follow through at home, work or social situations. Future studies should examine if interventions that improve pain or fatigue can also improve perceived function and physical performance.

## Abbreviations

5TSTS: Five Time Sit to Stand
6MWT: Six-Minute Walk Test
ACR: American College of Rheumatology
BMI: Body mass index
FAST: Fibromyalgia Activity Study with TENS
FIQR: Fibromyalgia Impact Questionnaire Revised
FIQR-Function: Fibromyalgia Impact Questionnaire Revised - Function Subscale
ICC: Intraclass correlation coefficient
MAF: Multidimensional Assessment of Fatigue
MAF-ADL: Multidimensional Assessment of Fatigue - Activities of Daily Living Subscale
NRS: Numeric Rating Scale
PCS: Pain Catastrophizing Scale
PROMIS: National Institutes of Health Patient Reported Outcome Measurement Information System
SD: Standard deviation
SEM: Standard error of measure
SF-36: Self-Report of Health Status
SF-36-PF: Self-Report of Health Status Physical Function Subscale
TENS: Transcutaneous Electrical Nerve Stimulation
TeP: Tender point
TSK: Tampa Scale of Kinesiophobia
VAS: Visual Analog Scale
